# Stereological estimates of dopaminergic, GABAergic and glutamatergic neurons in the ventral tegmental area, substantia nigra and retrorubral field in the rat

**DOI:** 10.1016/j.neuroscience.2008.01.046

**Published:** 2008-04-09

**Authors:** R.G. Nair-Roberts, S.D. Chatelain-Badie, E. Benson, H. White-Cooper, J.P. Bolam, M.A. Ungless

**Affiliations:** a Department of Zoology, University of Oxford, South Parks Rd, Oxford, OX1 3PS, UK; b Cardiff School of Biosciences, Cardiff University, Cardiff, CF10 3US, UK; c Medical Research Council Anatomical Neuropharmacology Unit, Department of Pharmacology, University of Oxford, Oxford, OX1 3TH, UK; d Medical Research Council Clinical Sciences Centre, Faculty of Medicine, Imperial College London, Hammersmith Hospital, Du Cane Rd, London, W12 0NN, UK

**Keywords:** VGluT2, GAD, reward, midbrain, basal ganglia, GAD, glutamic acid decarboxylase, HB, hybridization buffer, IFN, interfascicular subnucleus of the ventral tegmental area, IP, interpeduncular nucleus, lSN, lateral substantia nigra, PBP, parabrachial nucleus, PBS, phosphate-buffered saline, PBS-T, phosphate-buffered saline containing 0.1% Tween 20, PN, paranigral nucleus, RLi, rostral linear nucleus, RRF, retrorubral field, RT-PCR, reverse transcriptase–polymerase chain reaction, SN, substantia nigra, SNpc, substantia nigra pars compacta, TH, tyrosine hydroxylase, VGluT, vesicular glutamate transporter, vSN, ventral substantia nigra, VTA, ventral tegmental area

## Abstract

Midbrain dopamine neurons in the ventral tegmental area, substantia nigra and retrorubral field play key roles in reward processing, learning and memory, and movement. Within these midbrain regions and admixed with the dopamine neurons, are also substantial populations of GABAergic neurons that regulate dopamine neuron activity and have projection targets similar to those of dopamine neurons. Additionally, there is a small group of putative glutamatergic neurons within the ventral tegmental area whose function remains unclear. Although dopamine neurons have been intensively studied and quantified, there is little quantitative information regarding the GABAergic and glutamatergic neurons. We therefore used unbiased stereological methods to estimate the number of dopaminergic, GABAergic and glutamatergic cells in these regions in the rat. Neurons were identified using a combination of immunohistochemistry (tyrosine hydroxylase) and *in situ* hybridization (glutamic acid decarboxylase mRNA and vesicular glutamate transporter 2 mRNA). In substantia nigra pars compacta 29% of cells were glutamic acid decarboxylase mRNA-positive, 58% in the retrorubral field and 35% in the ventral tegmental area. There were further differences in the relative sizes of the GABAergic populations in subnuclei of the ventral tegmental area. Thus, glutamic acid decarboxylase mRNA-positive neurons represented 12% of cells in the interfascicular nucleus, 30% in the parabrachial nucleus, and 45% in the parainterfascicular nucleus. Vesicular glutamate transporter 2 mRNA-positive neurons were present in the ventral tegmental area, but not substantia nigra or retrorubral field. They were mainly confined to the rostro-medial region of the ventral tegmental area, and represented approximately 2–3% of the total neurons counted (∼1600 cells). These results demonstrate that GABAergic and glutamatergic neurons represent large proportions of the neurons in what are traditionally considered as dopamine nuclei and that there are considerable heterogeneities in the proportions of cell types in the different dopaminergic midbrain regions.

Ventral midbrain dopamine neurons play key roles in reward processing, learning and memory and movement (reviewed in Albin et al., 1989; Wise, 2004). In addition, their dysfunction is implicated in a number of disorders, including Parkinson’s disease (DeLong, 1990), schizophrenia (Goldstein and Deutch, 1992) and drug addiction (Hyman et al., 2006; Luscher and Ungless, 2006). They exert their influence through dense cortical and subcortical projections, most notably to the prefrontal cortex and basal ganglia (Swanson, 1982; Oades and Halliday, 1987; Albin et al., 1989). Ventral midbrain dopaminergic neurons are organized into three cell groups: retrorubral field (RRF) (A8), substantia nigra (SN) (A9), and ventral tegmental area (VTA) (A10), which, in addition to dopamine neurons, contain significant populations of GABAergic neurons (Nagai et al., 1983; Mugniani and Oertel, 1985; Kosaka et al., 1987). The GABAergic neurons subserve roles, both in the regulation of dopamine neuron activity through their local axon collaterals, and in the regulation of the activity of striatal and cortical neurons through their projecting axons (Bayer and Pickel, 1991; Van Bockstaele and Pickel, 1995; Carr and Sesack, 2000; Melis et al., 2002; Szabo et al., 2002; Korotkova et al., 2004). The possibility of the presence of GABA interneurons, however, cannot be excluded. Recent studies have also identified a population of glutamatergic neurons in the VTA that project to prefrontal cortex (Hur and Zaborszky, 2005). These glutamatergic neurons are a discrete population (i.e. they are negative for markers of dopamine and GABA (Yamaguchi et al., 2007), and are mainly located in rostro-medial VTA (Kawano et al., 2006; Yamaguchi et al., 2007)). Electrophysiological studies have shown that dopamine neurons typically have broad action potentials and slow firing rates (Grace and Bunney, 1983) compared with GABAergic neurons which have narrow action potentials and fast firing rates (Steffensen et al., 1998). More recently, neurons that have broad action potentials and slow firing rates but are non-dopaminergic have been described both *in vitro* (Cameron et al., 1997; Margolis et al., 2006) and *in vivo* (Ungless et al., 2004). One possibility is that these non-dopaminergic neurons are glutamatergic.

Although the numbers and distribution of dopamine neurons in the ventral midbrain have been quantified extensively (e.g. Swanson, 1982; German and Manaye, 1993; Harris and Nestler, 1996; Pioli et al., 2004), little information is known about the numbers and distribution of the populations of GABAergic and glutamatergic neurons. This type of quantitative data is critical for our understanding of the functional roles of the GABAergic and glutamatergic neurons and for our understanding of the functional roles of the midbrain dopamine nuclei. We therefore undertook a stereological study of neurons expressing a GABAergic marker (glutamic acid decarboxylase, GAD) and a marker of glutamatergic neurons (vesicular glutamate transporter 2 (VGluT2)) within major midbrain dopaminergic nuclei in the rat. Our approach was to perform *in situ* hybridization to detect mRNA for GAD and VGluT2, combined with immunohistochemistry for tyrosine hydroxylase (TH) to reveal dopamine neurons. This allowed us to clearly and reliably resolve individual cell bodies of defined phenotypes and then quantify them using unbiased stereology.

## Experimental procedures

### Animals

Ten male Sprague–Dawley rats, weighing approximately 250–300 g, were used in this study. Procedures were conducted in accordance with the Animals (Scientific Procedures) Act of 1986 (United Kingdom) and the Society of Neuroscience policy on the use of animals in neuroscience research. Every effort was made to use the minimum number of animals and to minimize suffering.

### Preparation of brains for *in situ* hybridization and immunohistochemistry

Animals were deeply anesthetized using a combination of ketamine (70 mg/kg) and medetomidine (0.5 mg/kg). They were transcardially perfused with a phosphate-buffered paraformaldehyde fixative (4% paraformaldehyde in 0.1 M phosphate buffer, pH 7.4). Brains were removed and post-fixed for 12 h in the same solution at 4 °C. They were sectioned in the coronal plane at 40 μm using a vibrating microtome (Vibratome 1500; Vibratome, St. Louis, MO, USA). Free floating sections were rinsed in phosphate-buffered saline (PBS), cryoprotected in a series of sucrose–ethylene glycol solutions (15% followed by 30% sucrose–ethylene glycol) and stored frozen in 30% sucrose–ethylene glycol at −20 °C until use. Solutions used for the preparation of brains and subsequent *in situ* hybridization steps were treated with an RNase inhibitor (0.1% DEPC).

### Riboprobes for *in situ* hybridization

Sense and antisense digoxigenin-labeled RNA probes for VGluT2, GAD 65 kDa isoform and GAD 67 kDa isoform were prepared using reverse transcriptase–polymerase chain reaction (RT-PCR) and *in vitro* transcription. cDNA transcript sequences were identified and downloaded from the Ensembl Rat Genome server (http://www.ensembl.org/Rattus_norvegicus/index.html). Clustal multisequence alignment analysis (Chenna et al., 2003) and Blast2-NCBI (http://www.ncbi.nlm.nih.gov/blast/) searches were used to identify 300–600 bp gene-specific regions within each transcript. Primers flanking target transcript regions were designed using the Vector NTI software package (Invitrogen). Details of primer sequences are shown in Table 1.

### RT-PCR

Two male Sprague–Dawley rats were deeply anesthetized with an overdose of sodium pentobarbital. The brains were carefully removed and the VTA, SN, striatum, cortex and cerebellum were rapidly dissected out. Tissue samples were snap-frozen in liquid nitrogen and stored at −80 °C until use. Frozen tissue samples were thawed in Trizol for total RNA extraction using Invitrogen’s Trizol system.

cDNA transcripts were produced from total RNA extracts using a reverse transcriptase (Superscript First-strand synthesis system, Invitrogen). Gene-specific cDNA sequences were selectively amplified from total cDNA preparations through PCR primed with the appropriate gene-specific primers (*Taq* polymerase PCR Kit, Qiagen). PCR products were analyzed on a 1% agarose gel. Comparison of actual product size with predicted size was used to establish that successful amplification of the desired target sequence had occurred.

### Synthesis of labeled riboprobes

PCR products were used as templates for riboprobe synthesis using an *in vitro* transcription kit (Digoxigenin RNA Labeling kit; Roche Diagnostics, UK). Labeled RNA products were subjected to alkaline hydrolysis in 0.1 M carbonate buffer (40 mM NaHCO_3,_ 60 mM Na_2_CO_3_) to yield approximately 100 bp length riboprobes.

### *In situ* hybridization

A 1:6 series of coronal sections (40 μm) through the midbrain was hybridized for GAD mRNA (both isoforms) (*n*=4) or VGluT2 mRNA (*n*=4). During sectioning, sections were collected in single wells. Eight to 10 free-floating sections encompassing an entire 1:6 series were combined in a single well for processing. Rostral–caudal order was re-established for mounting after processing by inspection of dopaminergic groups. The sections were washed several times in phosphate-buffered saline containing 0.1% Tween 20 (PBS-T) before transfer to hybridization buffer (HB, 50% formamide, 5× SSC, 100 μg/ml denatured sonicated salmon sperm DNA, 50 μg/ml heparin, 0.1% Tween 20) for preincubation (1 h at 65 °C). Denatured digoxigenin-labeled riboprobes (diluted in HB) were added to sections, with hybridization for 12 h at 65 °C. After a series of high to low stringency washes, sections were transferred to PBS for detection of DIG-labeled riboprobes using a mouse monoclonal anti-DIG antibody conjugated to alkaline phosphatase (Roche Diagnostics, UK; 1:2000 in PBS applied overnight at 4 °C). Sections were washed in PBS and transferred to a high pH buffer (HP, 100 mM NaCl, 100 mM Tris pH 9.5, 0.1% Tween 20, 50 mM MgCl_2_). A staining solution made up from NBT/BCIP (nitro blue tetrazolium chloride/5-bromo-4-chloro-3-indolyl phosphate) phosphatase substrate (Roche) diluted in HP was applied for 2.5 h. The phosphatase reaction was terminated by washing in PBS.

Optimal concentrations and incubation times were determined for each riboprobe in preliminary dilution series and time course experiments (results not shown). An incubation time of 2.5 h at room temperature was found to produce specific labeling of cell bodies in known GABAergic or glutamatergic cell regions, without significant background. *In situ* hybridization procedures were also carried out using sense DIG-labeled riboprobes to control for the occurrence of non-specific signals. No cellular labeling was observed in sections hybridized with sense riboprobes (results not shown).

### TH immunohistochemistry

Following *in situ* hybridization, sections were further processed to reveal TH immunoreactivity. The primary antibody was a sheep anti-TH from Chemicon (1:5000 in PBS-T with 3% normal rabbit serum, overnight at 4 °C), followed by a biotinylated secondary antibody (diluted 1:200, biotinylated rabbit anti-sheep, Vector Laboratories; Burlingame, USA) overnight at 4 °C. An avidin–biotin peroxidase enzyme complex was prepared and applied according to manufacturer’s instructions (Vectastain Elite ABC kit). Finally, sections were incubated for 5 min in a DAB/hydrogen peroxide substrate solution (prepared according to manufacturer’s instructions, Vectastain DAB substrate kit, Vector Laboratories). Sections were mounted in an aqueous mountant (Vectashield, Vector Laboratories) and coverslipped. Coverslips were fixed in place using clear nail polish. This TH antibody is widely used (e.g. Albéri et al., 2004; Schober et al., 2007) and only labels cells in dopaminergic regions. In control experiments where the primary or secondary antibody was omitted no labeling was observed. In addition, we have observed complete overlap between TH immunolabeling and *in situ* hybridization for TH mRNA (data not shown).

### Cell counting

Optical fractionator sampling was carried out on a Leica DMLB microscope equipped with a motorized stage and Lucivid attachment (40× objective). Sampling was implemented using the Stereoinvestigator software package (MicroBrightField Inc; Williston, USA). Midbrain dopaminergic groups were outlined on the basis of TH immunolabeling, with reference to a coronal atlas of the rat brain (Paxinos and Watson, 2005). Boundaries separating nuclei and subdivisions were identified based on the location of anatomical landmarks (e.g. fiber tracts), the neurochemical content of cells and regional variations in cell density, orientation and morphology (in line with descriptions in previous cytoarchitectonic studies, e.g. Phillipson, 1979a,b). For example, within the VTA the boundary separating dorsal PN (paranigral nucleus) from ventral PBP (parabrachial nucleus) was established based on visible changes in the density of dopaminergic cell bodies and their dendritic orientation. Cell bodies in PN tended to be more densely packed and with dendrites oriented in a ventromedial direction, in contrast with dopaminergic neurons in PBP, which were more sparsely distributed and appeared to lack any consistent dendritic orientation.

Total estimates of the number of GABAergic and dopaminergic neurons were obtained from four brains processed for GAD *in situ* hybridization and TH immunohistochemistry. Estimates of glutamatergic neurons were obtained from a further four brains processed for VGluT2 *in situ* hybridization and TH immunohistochemistry. In order for our counting to encompass the full rostro-caudal extent of the relevant midbrain dopamine nuclei, eight to 10 sections from a 1:6 series were analyzed for each brain. A random start was ensured by using a different well for the beginning of each series combined with the fact that the beginning of the collection of sections from the vibratome varied from brain to brain. Damaged/lost sections, of which there were few, were accounted for by the Stereoinvestigator software. Prior to beginning counting a section, average thickness was measured on a number of sections from different brains. There was no significant shrinkage from the original 38–40 μm thickness, as the sections were mounted in aqueous medium rather than dehydrated. A guard height of 8 μm was used with a sampling brick depth of 20 μm. Pilot studies were used to determine suitable counting frame and sampling grid dimensions prior to counting. Counting frame information: counting frame width (X) was 68.2 μm, height (Y) was 75 μm, depth was 20 μm. Guard regions of 8 μm thick were used for counting frame depth. Counting frame size was set so roughly 5 to 10 neurons were counted per frame and was maintained across all regions sampled. Sampling grid sizes were determined for different major regions (e.g. SN, VTA, and RRF) during pilot studies to allow approximately 15–20 sampling sites per region per section. Sampling grid size was equal for subnuclei within each major region. The sizes were: SN ((X) 366 μm, (Y) 276 μm); VTA ((X) 342 μm, (Y) 192 μm); RRF ((X) 351 μm, (Y) 251 μm). The software carried out these computations. Systematic random sampling was implemented by the Stereoinvestigator software until the designated region of interest was covered.

NBT-BCIP-positive *in situ*–labeled cells (blue) and DAB-positive immunolabeled cells (brown) were counted simultaneously within each counting frame position. A cell was only marked and counted if a nucleus surrounded by cytoplasm filled with a colored precipitate was clearly visible. Cells were counted only if they came into focus when racking the focus down through the sampling brick.

### Image capture and processing

Color digital images were acquired using a Leitz Dialux 22 microscope equipped with a Coolsnap color camera (Photometrics; Tucson, USA) and Openlab software (Improvision; Coventry, UK). Contrast and color balance were adjusted using Adobe Photoshop CS in order to include information-containing pixels and to reflect the true appearance of the tissue as far as possible. Line drawings illustrating the distribution VGluT2-positive neurons in individual sections were prepared from images exported from Stereoinvestigator, using Adobe Illustrator drawing software.

## Results

### General appearance of labeling

Both *in situ* hybridization and immunohistochemical reactions produced colored precipitates. In the NBT-BCIP *in situ* reaction, a blue–purple precipitate was deposited in the cell body of positive neurons. This blue–purple precipitate was clearly absent from the nucleus, with little or no product found in dendritic processes. Immunolabeling using the DAB-peroxidase system resulted in the formation of a brown precipitate within the cell body, axonal and dendritic processes. Although some light brown staining of the nucleus occurred, the intensity was always less than that seen in the cytoplasm allowing clear visualization of nuclei in labeled cells.

In a small number of VTA neurons, analysis at high magnification revealed the presence of both blue and brown precipitates, suggesting a positive signal for both *in situ* and immunohistochemical reactions. Thus, an average of approximately 0.58% of neurons examined in the VTA appeared to be double-labeled in GAD *in situ*/TH immunohistochemical preparations. This suggests that a small proportion of dopaminergic neurons in the VTA co-express GABAergic or glutamatergic markers as has been reported previously (e.g. see Gonzalez-Hernandez et al., 2001; Olson and Nestler, 2007 (GAD/TH colocalization) and Kawano et al., 2006 (VGluT2/TH colocalization)). However, the cell labeling methods used in this study are not suitable for an accurate quantitative analysis of double-labeled neurons as both the *in situ* and immunohistochemical reactions produced opaque precipitates that interfered with the detection of the other. An accurate analysis of the possible colocalization of TH, GAD, and VGluT2 will require the use of different cell labeling methods that utilize different markers e.g. fluorescence or autoradiographic labeling. For this reason, the data we present here deal only with cells that we could confidently identify as singly-labeled for GAD, TH or VGluT2. The small number of putative double-labeled cells encountered was excluded from the cell counting protocol.

### Dopaminergic (TH-positive) neurons

As has been shown extensively on previous occasions, immunohistochemical labeling for TH revealed significant populations of dopaminergic neurons within the midbrain nuclei (see Table 2). The total number of dopamine neurons contained within the three midbrain groups was approximately 71,000 (bilateral count). There were clear differences in the numbers of dopaminergic neurons among the different cell groups. The VTA contained approximately 40,000 dopaminergic neurons (bilateral count), the SNc nearly 25,000 (bilateral count) and the RRF approximately 6100 (bilateral count). There were also clear differences in the number of TH-positive neurons in different subdivisions within each dopaminergic nucleus (see Table 2). Within VTA, the PBP (parabrachial) and PIF (parainterfascicular) subnuclei contained the largest number of dopaminergic cells (bilaterally approximately 16,700 and 10,700 dopaminergic cells, respectively). In the SN, there were striking differences in the numbers of dopaminergic neurons in the subdivisions: the large majority of SN dopaminergic neurons were in the substantia nigra pars compacta (SNpc) subdivision with fewer in the lateral (lSN), ventral (vSN) and substantia nigra pars reticulata (SNpr) subdivisions.

### GABAergic (mRNA GAD-positive) neurons

**Fig. 1:**
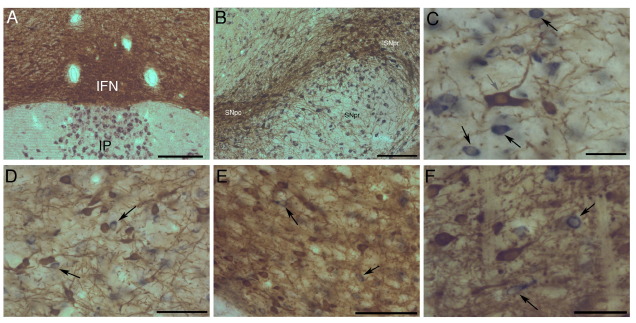
*In situ* hybridization for GAD (blue reaction product) combined with immunohistochemistry for TH (brown reaction product) in sections of the ventral midbrain. (A) GAD positive neurons in the IP ventral to the VTA. (B) Low-power micrograph of the nigral complex. Large numbers of GAD-positive neurons are present in the SNpr but also among the densely distributed TH-positive neurons in the SNpc and pars lateralis (lSN). (C) Higher-power image of the SNpr showing the clear differentiation between GAD-positive neurons (black arrows) and TH-positive neurons (gray arrow). Note that the *in situ* labeling is confined to the perikaryon, whereas the TH immunolabeling also extends into dendritic processes. (D) High-power image of the RRF showing GAD-positive neurons (black arrows) among the brown TH-positive neurons. (E, F) High-power images of subdivisions of the VTA (E: paranigral VTA; F: parabrachial). Some of the GAD-positive neurons are indicated by black arrows. Scale bars=200 μm (A, B); 100 μm (D, E); 50 μm (C, F).

The *in situ* hybridization revealed a number of distinct populations of GABAergic neurons spread across the midbrain (see Table 2). GAD-positive labeling was particularly prominent in the interpeduncular nucleus (IP) (Fig. 1A) and the SNpr (Fig. 1B, C). In the VTA and SNpc, a smaller contingent of GAD-positive neurons was observed scattered throughout the dense TH-positive cell populations (Fig. 1E, F). While GAD-positive cell bodies were observed infrequently in the interfascicular subnucleus of ventral tegmental area (IFN), a higher density occurred in lateral SNPc (SNl) and RRF (Fig. 1D). The estimates of absolute numbers of GAD-positive neurons in the major midbrain dopaminergic nuclei are shown in Table 2.

In addition to differences in absolute numbers of GAD-positive neurons between different midbrain nuclei, because of the differences in numbers of TH-positive neurons the relative proportions of different classes of neurons varied (Table 2). The largest proportion of GAD-positive neurons relative to TH-positive neurons was in the SNpr, where approximately 70% were GAD-positive and 30% TH-positive. On the other hand, the smallest proportion of GABAergic cells was in the SNpc, where 29% of neurons were positive for GAD.

Differences in the relative numbers of GAD-positive neurons were also seen among the different subnuclei (Table 2). Thus, in VTA the greatest proportion of GAD-positive neurons (relative to TH neurons) was found in the PIF subdivision, and the lowest in the IFN. As a general trend, the proportion of GABAergic neurons relative to dopaminergic neurons was greater in more caudal VTA subnuclei (PIF, PN), as compared with rostral VTA areas (rostral linear nucleus (RLi), IFN). Variations in the relative numbers of GABAergic neurons among the subnuclei of the SN were even more striking: GAD-positive neurons in SNpc were outnumbered by TH-positive neurons by a factor of about 2.5:1. In SNpr, vSN and lSN the situation was reversed: GAD-positive neurons in these areas outnumbered TH-positive neurons by factors of 9:1, 1.3:1 and 2:1 respectively.

### Glutamatergic (VGluT2-positive) neurons

**Fig. 2:**
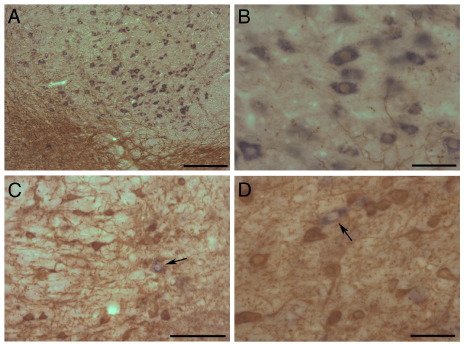
*In situ* hybridization for VGluT2 (blue reaction product) combined with immunohistochemistry for TH (brown reaction product). (A) VGluT2-positive neurons in the red nucleus located dorsal–medial to the VTA, which is heavily immunostained for TH. (B) Higher-power image of VGluT2-positive neurons in the red nucleus (note the occasional TH-positive process coursing among the glutamatergic neurons). (C) A VGluT2-positive neuron (black arrow) in the RLi of VTA. (D) A VGluT2-positive neuron (black arrow) in the parabrachial region of the VTA (PBP). Scale bars=200 μm (A); 100 μm (C); 50 μm (B, D).

**Fig. 3:**
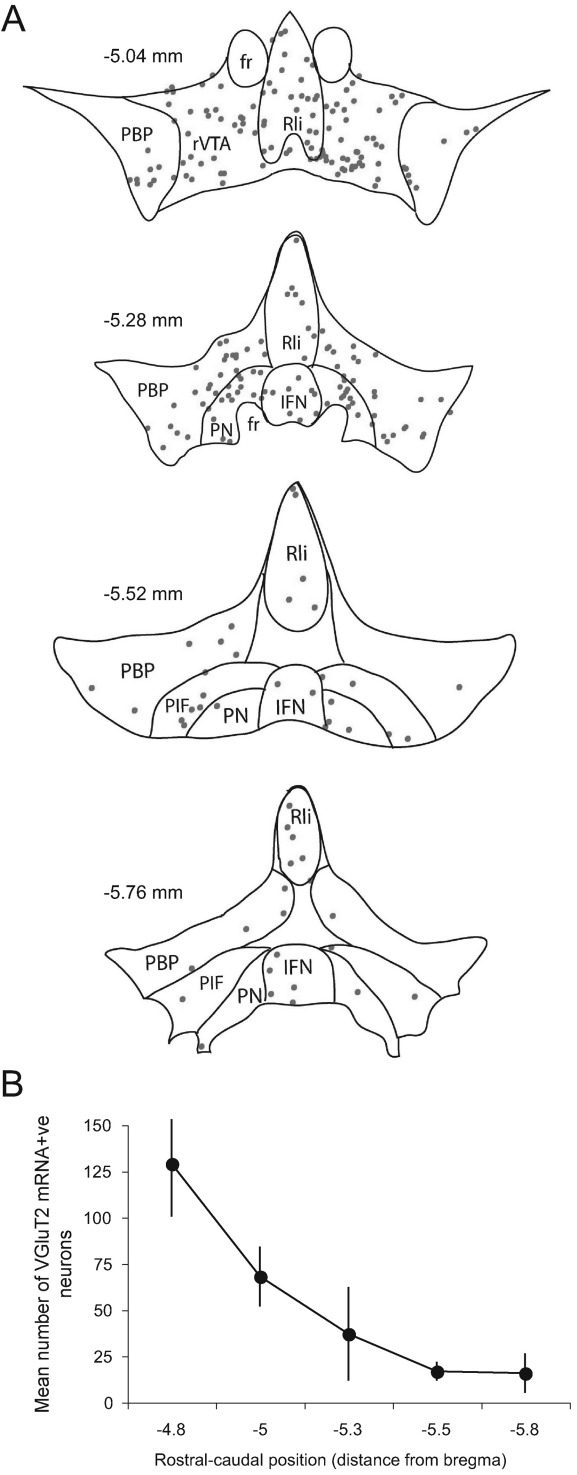
Distribution of VGluT2-positive neurons in the VTA. (A) Blue dots indicate the located of single VGluT2-positive neurons in a single animal. Schematics are derived from the atlas of Paxinos and Watson (2005); the distance from bregma is indicated on the left of each plot. (B) Graph illustrating the decrease in the number of VGluT2-positive neurons counted per section going from rostral to caudal regions of the VTA (data obtained from four animals, error bars represent standard error of the mean). For interpretation of the references to color in this figure legend, the reader is referred to the Web version of this article.

The *in situ* hybridization data revealed that VGluT2-positive cell bodies were far less abundant in the midbrain than either TH-positive or GAD-positive neurons. Midbrain VGluT2-positive labeling was especially prominent in the red nucleus (RN), located dorsal to VTA (Fig. 2A, B) and the medial terminal nucleus of the optic tract (not shown). However, few if any VGluT2-positive neurons were observed in the SNpr, SNpc and RRF. On the other hand, a clearly-labeled, though relatively small, population of VGluT2-positive cells was apparent in VTA (Fig. 2C, D). Our quantitative analyses show that VGluT2-positive neurons in the VTA account for 1600 neurons on average (1599±94.4, mean±standard error of the mean; bilateral estimate obtained from four animals), which represents only 2–3% of all neurons in the VTA. The VGluT2-positive neurons displayed a far more restricted regional distribution than TH-positive or GAD-positive neurons. Thus, most of VGluT2-positive neurons were located in the rostral parts of the VTA and their numbers decreased sharply in caudal VTA (Fig. 3A, B). A higher density of VGluT2-positive neurons was present in medial versus lateral portions of VTA: VGluT-positive cells were relatively enriched in the medial subdivisions (rostral VTA, RLi, PN and medial PBP; Fig. 3A, B).

## Discussion

Quantitative aspects of the numbers and distribution of dopamine neurons in the VTA, SN and RRF have been widely studied, however, it is becoming increasingly evident that within these nuclei are populations of non-dopaminergic neurons that are of functional importance. We have therefore undertaken simultaneous quantitative analyses of dopaminergic, GABAergic and glutamatergic neuronal populations in the VTA, SN and RRF using unbiased stereology. This simultaneous analysis revealed considerable variation in the relative numbers of dopaminergic and GABAergic neurons at regional and subregional levels. In general, we found that there are more GABAergic neurons than previously assumed. Our data also provide the first unbiased stereological count of the glutamatergic neurons in the VTA.

### Comparison of our data to previous estimates

One other quantitative study of the total number of neurons in the SN has been undertaken using unbiased stereology (Oorschot, 1996). Their reported estimate (unilateral count) of the total number of neurons in the A9 group was 33,500 which is in close agreement with the figure of 33,060 that we estimate for the total cell population contained in all subdivisions of A9 (SNPc, SNPr, lSN, vSN). Other studies have concentrated almost exclusively on dopamine neurons, and generally they report lower numbers of dopaminergic neurons than we do (e.g. Swanson, 1982; German and Manaye, 1993; Harris and Nestler, 1996). For example, in the VTA using total cell counts (with Abercrombie’s correction) Swanson (1982) found a total of 14,000 (unilateral estimate) of which two-thirds were dopaminergic (TH-immunoreactive) and one-third non-dopaminergic. In spite of the difference in absolute estimates, our estimate that 35% of all cells in VTA are GABAergic agrees fairly well with Swanson’s estimate that at least one-third of VTA cells are non-dopaminergic.

In a more recent, computer-assisted study dopaminergic neurons labeled by immunohistochemistry were counted in the same midbrain cell groups as those studied here: A8, A9 and A10 in the rat (German and Manaye, 1993). Bilateral cell counts obtained were: A8, 2600, A9, 21,000, A10, 20,400. While our population estimate for the A9 group (SNPc) is similar to their estimate (25,000 bilateral), our population estimates for VTA and RRF are higher (RRF: 6100 vs. 2600; VTA 40,000 vs. 20,400). These, and other, studies did not typically use unbiased sampling methods, and other features such as computer-assisted counting may have resulted in the lower numbers reported. We believe it is likely that our study provides a more accurate estimate because we used unbiased optical fractionator sampling (for discussion of the merits of unbiased stereology see Howard and Reed, 1998).

### GABAergic neurons in the VTA, SN and RRF

Few formal attempts have been made to quantify the populations of GABAergic neurons within the VTA, SN and RRF (also reviewed in Kalivas, 1993; e.g. Ford et al., 1995). In general, they report lower figures than ours (1090 for RRF, 1080 for SNPc and 1200 for VTA; Ford et al., 1995), a difference that is likely to be related to the use of immunohistochemical methods with antibodies against GAD. Our choice of *in situ* hybridization for GAD mRNA to identify GABAergic cells stemmed from previous difficulties we and others encountered in resolving significant numbers of immunolabeled cell bodies in midbrain nuclei using a number of commercially available antibodies against GAD (Nair-Roberts, Chatelain-Badie, Bolam and Ungless, unpublished observations). We believe, therefore, that our results provide a more accurate account of the GABAergic population. In addition we have presented an analysis by subregion that was previously lacking. Overall, our data suggest that the quantitative importance of GABAergic neurons relative to dopaminergic neurons within the VTA, SN and RRF has been underestimated in previous studies. GABAergic neurons thus represent a large proportion of neurons in dopaminergic structures in the ventral midbrain and are likely to be critically involved in the regulation of the activity of dopaminergic neurons and the output of these nuclei.

### Glutamatergic neurons in the VTA

The existence of a distinct population of glutamatergic neurons identified on the basis of VGluT2 *in situ* hybridization has recently been described (Kawano et al., 2006; Yamaguchi et al., 2007). Our identification of, and estimate of the total number of the glutamatergic neurons in VTA is thus in support of these findings. Furthermore, the regional distribution of the VGluT2 cells observed in our study (Fig. 3A, B) is consistent with the findings of Yamaguchi et al. (2007).

We found that the VTA (bilateral) contains about 1600 neurons (i.e. 2–3% of the total population of neurons in the VTA). Although this is a small absolute number and a small proportion of the total population, they were highly enriched in medial parts of rostral VTA, with a density that tailed off very rapidly in more caudal and lateral areas. Little is known about these neurons, except that they project to medial prefrontal cortex and somatosensory cortex (Hur and Zaborszky, 2005). The existence of a neurochemically-distinct rostral VTA cell group may be relevant to the functional differences between rostral and caudal subdivisions of VTA (e.g. Carlezon et al., 2000; Olson et al., 2005). It is important to characterize this population of glutamatergic neurons and relate them to other identified populations of non-dopaminergic neurons (Cameron et al., 1997; Ungless et al., 2004).

*In vitro* slice studies have demonstrated that stimulation of the VTA evokes glutamatergic excitatory synaptic currents (EPSCs) in the nucleus accumbens (NAc) (Chuhma et al., 2004). These experiments, and others, have been taken to support the idea that dopaminergic neurons co-release glutamate and dopamine (Sulzer et al., 1998; Joyce and Rayport, 2000; Chuhma et al., 2004). An alternative possibility is that the separate population of non-dopaminergic glutamatergic neurons in the VTA is responsible for fast excitatory transmission in this pathway. Furthermore, although a small proportion of dopamine neurons have been reported to express mRNA for VGluT2 in one study (Kawano et al., 2006), the findings of Yamaguchi et al. (2007) suggest that dopaminergic neurons in the SN/VTA do not express VGluT2. We also found very few neurons that co-express TH and VGluT2, but as discussed in the Results section our methods were not well-suited to assess co-localization.

## Conclusion

Our detailed quantitative data relating to dopaminergic, GABAergic and glutamatergic neurons in the VTA, SN and RRF lay an essential foundation for future functional insights into the principles of operation of the microcircuits of these regions and their interactions with other regions of the brain. Resolving a complete picture of local and global neurochemical interactions in the midbrain dopamine nuclei will require the investigation of the function of individual identified cell types, their interaction with other cell types and their projections.

## Figures and Tables

**Table 1:** Primer sequences

Isoform	Sequence
GAD65	
Forward	5′-CTGTAATACGACTCACTATAGGGCACTTCTCCACGCAACAGACC-3′
Reverse	5′-ACTGAATTAACCCTCACTAAAGGCGCTGTCTGTTCCGATCCCC-3′
GAD67	
Forward	5′-ACTGTAATACGACTCACTATAGGGGCAGGCCTCCAAGAACCT-3′
Reverse	5′-ACTGAATTAACCCTCACTAAAGGAGCCCCATCACCGTAGCAACC-3′
VGlut 2	
Forward	5′-GCGCTAATACGACTCACTATAGGGGATTTGGTTGCGTTAAGAC-3′
Reverse	5′-GCGCAATTAACCCTCACTAAAGGCGGTTATCCTGCTTCTTCTC-3′

**Table 2:** Stereological estimates of the numbers of TH-immunopositive neurons and GAD mRNA-positive neurons in the VTA, SN and RRF (bilateral counts)

Nucleus	Subnucleus	TH	GAD	TH/GAD ratio
RRF		6163±1562	8541±1923	0.72±0.08
VTA		40174±5315	21011±2453	1.92±0.18
	PBP	16435±4178	7047±2071	2.41±0.23
	IFN	3475±386	464±35	7.47±0.52
	Rli	3310±801	1556±560	3.24±1.22
	PN	6202±499	3167±254	2.01±0.25
	PIF	10752±1701	8777±1711	1.28±0.11
SN		24906±4209	41214±4268	0.60±0.06
	Pc	15772±3160	6400±1545	2.60±0.28
	Pr	2430±728	21657±3544	0.11±0.02
	V	2941±670	4814±1370	0.73±0.14
	L	3763±539	8343±867	0.46±0.06

Data obtained from four animals; values are means±S.E.M. Eight to 10 sections from a 1 in 6 series were analyzed per brain.
